# Comparison of target-controlled infusion of sufentanil and remifentanil in blunting hemodynamic response to tracheal intubation

**DOI:** 10.5249/jivr.v5i2.325

**Published:** 2013-06

**Authors:** Naser Yeganeh, Bahman Roshani, Hossein Latifi, Afshin Almasi

**Affiliations:** ^*a*^Department of Anesthesiology, Imam Reza Hospital, Kermanshah University of Medical Sciences, Kermanshah, Iran.

**Keywords:** Injury, Intravenous analgesics, Sufentanil, Remifentanil, Tracheal intubation, Target-controlled infusion

## Abstract

**Background:**

Maintaining blood pressure (BP) and heart rate (HR) after laryngoscopy and tracheal intubation has always been a concern in injured patients. Opioids can attenuate or stop an increase in these two parameters if administered with proper doses or targets in target-controlled infusion (TCI). Remifentanil and sufentanil are widely used for this purpose because their cardiac side effects are low and, especially in traumatic patients, they are tolerated well. A comparison of the benefits and limitations of these two opioids in TCI is much needed. A literature review in electronic data bases revealed few results.

**Methods:**

40 normotensive patients were enrolled to this randomized clinical trial study. After BIS guided anesthesia with a target-controlled propofol infusion and muscle relaxation with cisatracurium, remifentanil and sufentanil were infused using TCI with 2 and 0.2 ng.ml-1 targets respectively. BP and HR were recorded for five data points and compared with Fischer's exact test.

**Results:**

Systolic, mean and diastolic arterial pressure and HR in different points of the study remained below baseline values but were out of control in some cases, however the out-of-control values showed significant difference between the two groups only for heart rate changes. The relative risk for producing out-of-control changes with remifentanil compared to sufentanil is significantly more than 1 for HR decrease.

**Conclusions:**

Sufentanil produced more common pre-intubation hypotension than remifentanil in propofol anesthetized patients but this hypotension disappeared sooner than remifentanil after tracheal intubation. Both opioids prevent an increase in BP and HR after tracheal intubation but the side effects (hypotension and bradycardia) may be a cause for concern (IRCT138710011361N3).

## Introduction

Blood pressure and heart rate are among the most widely used hemodynamic parameters during anesthesia and surgery to evaluate cardiovascular status and in traumatic injured patients to preserve these hemodynamic parameters in physiological ranges are particularly important. Although there are also other parameters such as stroke volume, cardiac index and systemic vascular resistance which are more important for this evaluation, they have more limited use in daily clinical anesthesia practice; hence BP and HR are the clues for further cardiovascular assessment during anesthesia. Tracheal intubation under light anesthesia often causes a hemodynamic response probably generated by direct laryngoscopy which may be even more intense than skin incision. ^1^ On the other hand, preserving blood pressure and heart rates within a narrow range is a concern for anesthesiologists before induction of anesthesia especially in patients with limited cardiac reserves. Various drugs and techniques have been investigated for the blunting of these stimuli and the bringing about of catecholamine release. ^2,3,4^ Opioids are always among the drugs of choice for this purpose. ^5^ Many opioids such as fentanil, alfentanil, sufentanil and remifentanil have an important role in controlling changes in hemodynamic variables in response to tracheal intubation. ^3,6,7^ Remifentanil on the other hand is one of the newest synthetic opioids with unique characteristics which makes it ideal for the per-induction period in anesthesia mainly a short onset and the fastest offset among anilidopiperidine family,^8,9^ however conventional administration of these opioids should be timed so their maximum effects are predictably matched with the stimuli. In this regard sufentanil and fentanil may have slower effect site equilibration times than alfentanil ^3^ and remifentanil but when a target-controlled infusion technique is used for administration it is the physician's decision which determines the rapidity of equilibration. 

Several authors have compared the blunting of the hemodynamic response to tracheal intubation when using manual administration of different opioids ^6,10,11,12,13^ however comparison of target-controlled infusion (TCI) of remifentanil and sufentanil which are technically available in commercial TCI pumps is also necessary because of the frequent use of these two opioids in daily anesthesia practice. Hence we believe our study is novel and constitutes the first comparison of these two drugs for this goal.

## Methods

Participants enrolled in the study from January 2008 until July 2010. After approval from the institute’s ethical committee and written informed consent from each patient we enrolled 40 normotensive patients to this randomized double-blinded clinical trial study. Inclusion criteria were ASA class I-II patients, aged from 18 to 60 years old who were candidates for general orotracheal anesthesia to perform non -emergent abdominal surgeries in traumatic patients. Exclusion criteria were: age below 18 and above 60 years; treated or untreated hypertension; gross upper airway anomaly; previous sensitivity to propofol, egg or soya bean; chronic use of alcohol, sedatives or opioid abuse; and predicted difficult intubation according to preoperative assessment clinic sheet. Also, every patient with a laryngoscopy duration of more than 15 seconds or several previous laryngoscopies was then excluded from the study. All patients were premedicated with oral diazepam 5 mg the night before the operation. In the preoperative period the patients were visited again by the anesthesia resident and after review of their medical history and brief examination, the height and weight of patients were measured and recorded in the checklist. In the operating room a 20-gauge intravenous catheter was inserted in a large antecubital vein and 10ml.kg-1. Ringer solution was infused slowly during a 30 minute period. The Ethical Committee did not allow the use of any invasive monitoring such as intra-arterial catheter in the absence of other clinical indications; hence standard monitoring such as 3-lead electrocardiogram, noninvasive blood pressure and pulse oximetrey were applied. The monitoring unit used for study was DATASCOPE (model passport II, USA) and the same unit was used for all measurements in this study. The module of NIBP of the monitoring unit was calibrated by the medical engineering department of the hospital before the first case enrollment. One patient in R group was excluded from the study because of propofol induced hypotension after 14 minutes of steady propofol infusion (more than 15% decrease in systolic and mean blood pressure) and one patient in S group was also excluded because of a laryngoscopy duration of more than 15 seconds. The next patients on the list were substituted in each group. In 5 patients interval recalibration was done and if there were more than 5 mmHg differences with standard pressure all previous 5 cases were excluded. Baseline (pre-induction) blood pressure and heart rate of the patients were measured and recorded after the Ringer serum infusion was ended; this was the To point which was assumed as the baseline to compare the consecutive changes of BP and HR. The skin of the patient’s forehead was scrubbed, cleaned and prepared with alcohol and gauze, and then a four electrode BIS sensor was attached to the forehead according to instructions. On connecting the sensor to the converter of the BIS monitor (Aspect medical system, A-2000 monitor version 3.23 USA, Newton, MA) a measurement of level of consciousness was started. The inside of the electrodes was applied with gel only if the recorded impedance showed more than 10kΩ. The smoothening time of the BIS monitor was set as 15 seconds. Propofol, sufentanil and remifentanil were administered with a Frenesius Modular DPS infusion pump connected to Base Prima with integrated Orchestra TCI system (Fresenius infusion system, France). The patients and physician responsible for data collection were blind to study groups. Propofol (Fresenius Kabi Company, Germany) infusion was started first with the Schnider^14^ three compartment model and an effect site concentration target of propofol (CeP) as 3μg.ml-1, with induction time set as 120 seconds and maximum plasmatic concentration of propofol limited to 6μg.ml-1. The goal of slow induction was the modest reduction in blood pressure produced with propofol.

After equilibration of predicted and set target concentrations, if the BIS value was above 60, CeP was increased 0.5μg.ml-1 and if the BIS value was below 45 the CeP was reduced 0.5μg.ml-1 until the BIS value was located between 45 and 60. As new concerns are emerging about prolonged depression of the cardiovascular system after induction of general anesthesia with propofol^15^ we tried to maintain the patients in a physiologically steady state before performing the experimental intervention; hence we allowed the patients to stay in steady effect site concentration after stable BIS value for 14 minutes to reach physiologic stability. In this period the patients were ventilated with a mask and pure oxygen. According to a randomized numbers sequence, patients were assigned to one of two groups; sufentanil(S) or remifentanil(R) group.

After reaching the hypnotic steady state, BP was measured and if it differed more than 15% from the T0 value, the appropriate treatment was done and the patient was excluded because the propofol induced hypotension might confound the result of our intervention, if not the study was continued and cis-atracurium bromide (GlaxoSmithKline, UK) was administered bolus i.v. with a dose of 0.15 mg.kg-1 of body weight and remifentanil (GlaxoSmithKline, UK) with effect site concentration (CeR) of 2 ng.ml-1 with Minto ^16^ three compartments model in group R or sufentanil (Janssen-CILAG; Janssen pharmaceutical N.V., Belgium) with effect site concentration (CeS) of 0.2 ng.ml-1 with Gepts ^17^ three compartments model in group S were infused by the other modules of the same TCI pump performing propofol infusion. The selected targets for remifentanil and sufentanil are equipotent targets and suitable for suppressing major surgical stimulus. ^18-20^ After 2 minutes and following confirmation of one single twitch on train-of-four stimulation (diagnosed by neuromuscular monitoring) and equilibration of predicted and set target concentration of opioids in each group, BP and HR were measured, recorded and noticed as T1 (pre-intubation) point. Laryngoscopy and tracheal intubation were performed using standard Macintosh blade laryngoscope size 3 or 4 (Heine, USA) and then a cuffed tracheal tube size 7.0 or 7.5 (Mallinkrodt, Ireland) was placed in the trachea. The laryngoscopist was the same for all patients. Variables of the study were measured and recorded immediately (T2 point), 1 minute, 3 minutes and 5 minutes (T3, T4 and T5 points) after tracheal intubation. After recording of the variables of the study, anesthesia continued according to the patient’s status and with decision taking by the anesthesiologist. Independent t-test was used to compare mean BIS values and CeP in T1 points between R and S groups.

A relevant change in the BP was assessed as a 15% change in systolic, diastolic and mean arterial pressure and 10% for heart rate with a 90% chance to detect a difference, a confidence interval of 95% and type 1 error (α) = 0.05. Hence the sample size was assessed as 33 cases which we enrolled 40 cases in the study. Every missed or excluded patient was substituted with the next patient (intention to treat). If the changes of variables in T2, T3, T4 and T5 points compared to T0 point (baseline) were in the range of 15% and 10% for blood pressure and heart rate respectively, they were assumed as being in control and if the changes were more than these ranges they were assumed as out of control. Fisher’s exact test was used to compare the changes in different data points in each group. The relative risk for out-of-control values of blood pressure and heart rate for remifentanil compared to sufentanil in different data points was assessed and a risk of more than 1 was confirmed as significant if the p value was lower than 0.05. This study was primarily registered in UMIN (Japan) with number UMIN000001564 in 2008/12/30 (www.umin.ac.jp) and also in IRCT with number IRCT138710011361N3 in 2008/12/12 (www.irct.ir).

## Results

Forty patients completed the study without need for treatment with vasodilators, vasopressors or atropine hence variables were measured and gathered in 240 data points totally. Characteristics of the patients in the two groups are shown in Table 1. Calibration of the NIBP module in 5 patients’ intervals showed acceptable results confirming reliability of BP measurements. The mean BIS values in T1 point (47.3±2.8 in S group vs. 50.2± 1.9 in R group with P value 0.15) showed sufficient and comparable depth of BIS guided propofol hypnosis in two groups before the laryngoscopy, this was accordant with comparable mean CeP in two groups (4.11±0.92 μg.ml-1 in S group vs. 4.20±0.85 μg.ml-1 in the R group with P value=0.32) in T1 point.

**Table 1:** Patients' characteristics in two study groups

Characteristics	Remifentanil	Sufentanil	P
Age(y)	31.3±8.4	33.5±6.3	>0.05
Sex(M/F)	11/9	12/8	>0.05
Weight(kg)	70.2±14.8	75.9±18.4	>0.05
Height(m)	1.69±9.31	1.75±11.26	>0.05
BMI(kg/m²)	24.9±5.3	26.5±4.8	>0.05
BSA(m²)	1.85±0.25	1.77±0.20	>0.05
ASA(I/II)	18/2	17/3	>0.05
BIS(T1)	50.2±1.9	47.3±2.8	>0.05
BP(T0)(S/M/D)	130±11.2/95±9.8/78±7.6	129±13.6/99±10.9/83±10.3	>0.05
HR(T0)	93±11	92±9	>0.05

BMI: body mass index, BSA: body surface area, ASA: classification of American society of anesthesiologists, BIS: bispectral index, BP: blood pressure, HR: heart rate, T1: time point 1, T0: time point 0. Significant differences between two groups were not seen (P >0.05) in characteristics S:systolic, M:mean,D:diastolic blood pressure

**Figure 1:**
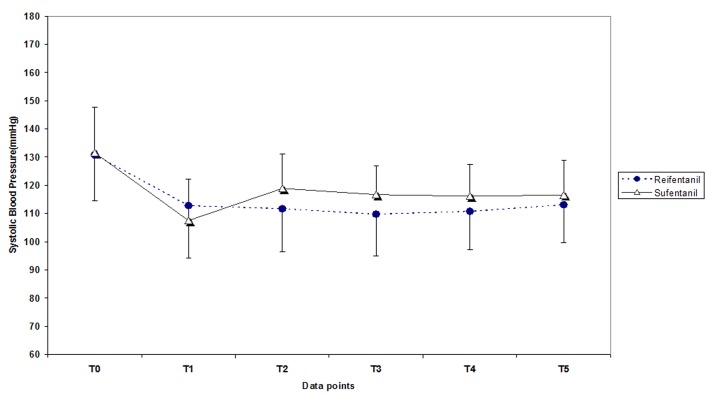

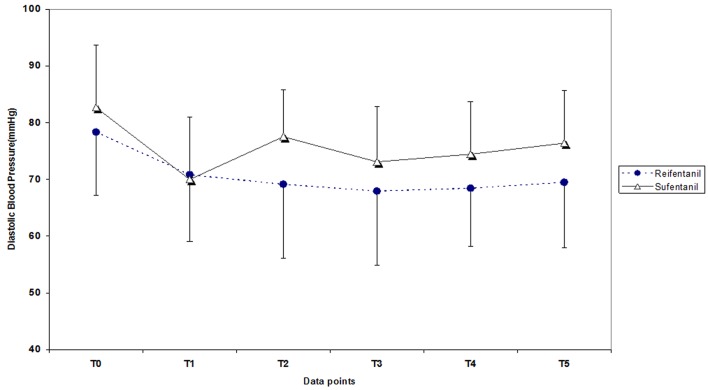

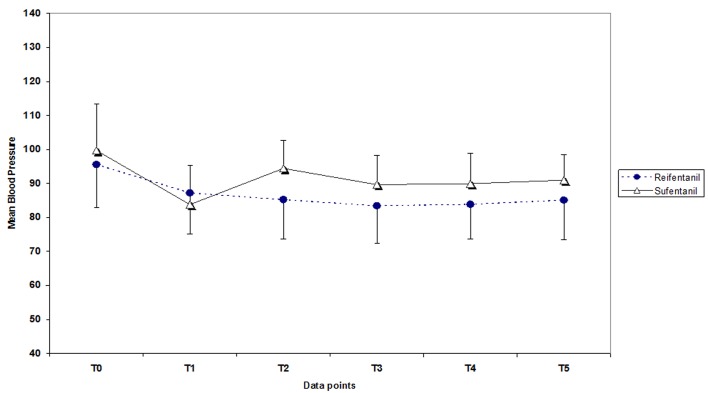
Systolic, diastolic and mean arterial pressure in remifentanil and sufentanil groups in different data points. T0: pre-induction, T1: pre-intubation, T2: immediately after, T3: 1 minute, T4: 3 minutes and T5: 5 minutes after tracheal intubation.

**Figure 2:**
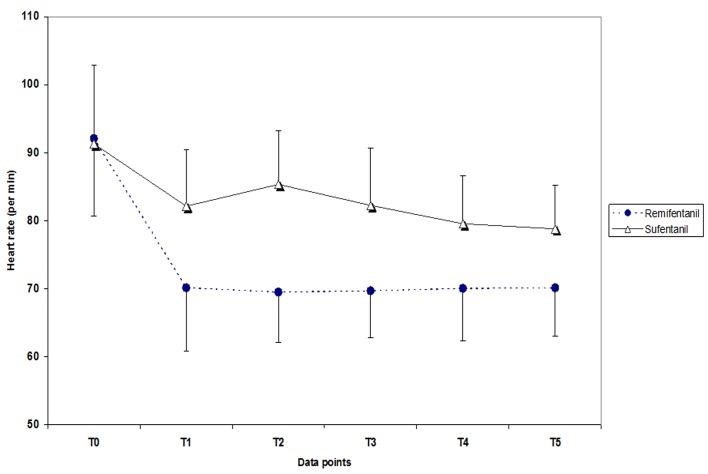
Heart rate in remifentanil and sufentanil groups in different data points. T0: pre-induction, T1: pre-intubation, T2: immediately after, T3: 1 minute, T4: 3 minutes and T5: 5 minutes after tracheal intubation.

Systolic, mean and diastolic arterial pressure and heart rate in different points of the study remained below baseline values but were out of control in some cases which are shown in Figure 1 and 2, however the out-of-control values showed significant differences between the two groups only for heart rate changes. Also assessment of in control and out-of-control changes of the variable in T2, T3, T4 and T5 compared to T0 and relative risk for producing of out-of-control changes for remifentanil compared to sufentanil are shown in Table 2.

**Table 2:** Assessment of relative risk for out of control values of blood pressure and heart rate producing by remifentanil compared to sufentanil in different data points compared to T0 (pre-induction).

	Remifetanil		Sufetanil		P value	Relative Risk
	In control	Out of control	In control	Out of control		Out of control Remi/Suf
SBP						
T1-T0	50%(10)	50%(10)	80%(16)	20%(4)	0.09	0.62
T2-T0	65%(13)	35%(7)	45%(9)	55%(11)	0.34	1.44
T3-T0	65%(13)	35%(7)	55%(11)	45%(9)	0.74	1.18
T4-T0	70%(14)	30%(6)	55%(11)	45%(9)	0.51	1.27
T5-T0	65%(13)	35%(7)	55%(11)	45%(9)	0.74	1.18
DBP						
T1-T0	15%(3)	85%(17)	25%(5)	75%(15)	0.69	0.60
T2-T0	25%(5)	75%(15)	15%(3)	85%(17)	0.69	1.66
T3-T0	40%(8)	60% (12)	20%(4)	80%(16)	0.30	2.00
T4-T0	35%(7)	65%(13)	25%(5)	75%(15)	0.73	1.40
T5-T0	30%(6)	70(14)	15%(3)	85%(17)	0.45	2.00
MBP						
T1-T0	25%(5)	75%(15)	45%(9)	55%(11)	0.32	0.55
T2-T0	45%(9)	55%(11)	15%(3)	85%(17)	0.08	3.00
T3-T0	35%/(7)	65%(13)	40%(18)	60%(2)	0.99	0.87
T4-T0	50%(10)	50%(10)	30%(6)	70%(14)	0.33	1.66
T5-T0	35%(7)	65%(13)	25%(5)	75%(15)	0.73	1.40
HR						
T1-T0	85%(17)	15%(3)	55%(11)	45% (9)	0.08	1.54
T2-T0	85%(17)	15%(3)	40%(8)	60%(12)	0.00*	2.12*
T3-T0	90%(18)	10%(2)	55%(11)	45%(9)	0.03*	1.63*
T4-T0	95%(19)	5%(1)	65%(13)	35%(7)	0.04*	1.46*
T5-T0	80%(16)	20%(4)	65%(13)	35%(7)	0.48	1.23

*Relative risk more than 1 is significant if p value <0.05.

## Discussion

In this study both sufentanil and remifentanil controlled blood pressure increase after laryngoscopy with TCI but they decreased systolic, diastolic and mean arterial pressure in some cases. Opioids are the most useful and widely accepted drugs which are administered as an adjuvant by anesthesiologists before laryngoscopy and tracheal intubation to prevent blood pressure increase but they may decrease blood pressure if the dose or concentration is chosen inappropriately, the condition which is synergistically intensified with hypnotic agents. ^2^ As we didn’t find any target controlled comparison of these two opioids for tracheal intubation responses in the literature review we chose sufentanill and remifentanil targets which were high enough to be effective for preventing blood pressure increase. Indeed opioids can attenuate or stop increase or may decrease blood pressure when administered in a dose- dependent fashion.^18^ In our study when remifentanil or sufentanil were administered a decrease in systolic blood pressure was more common than the mean and as was the case with diastolic blood pressure but the time of hypotension differed in the two groups: when sufentanil was administered hypotension was more common in the pre-intubation period and when remifentanil was administered it was more common in the three minutes after intubation. 

As several studies have discovered that maximum heamodynamic responses occur about 1-5 minutes after tracheal intubation, ^2,4,19^ we measured blood pressure and heart rate from commencement until 5 minutes after completion of tracheal intubation for more confidence but didn't find a specific time point for maximum responses, indeed we didn’t find any exaggerated responses because they were suppressed well by effect-site opioid concentration. We think this may be due to the difference between TCI and other conventional administration methods. Time to peak effect of intravenous analgesics should be spent when they are administered in conventional bolus form: this time is 5.6 min and 1.6 min for sufentanil and remifentanil respectively. ^20^ It's only after this period that appropriate effect site concentration is guaranteed and if tracheal intubation is performed sooner the goal of opioid administration is not achieved. However when TCI is used the pharmacokinetic driven model predicts the target in the effect site precisely and even the operator can determine the time of target equilibration in the effect site.

In a study similar to our own, but not in target-controlled manner, Iannuzzi^21^ and coworkers found that in normotensive patients administration of small doses of either remifentanil (0.1μg.kg-1.min-1) or sufentanil (0.01μg.kg-1.min-1) after target-controlled induction with propofol (3 μg.ml-1 effect site) was an effective strategy to blunt the cardiovascular response to intubation, but the sufentanil group showed more stable systolic and diastolic blood pressure vs. transient systolic and diastolic pressure variations in the remifentanil group. Also Safavi and coworkers^22^ in their study compared sufentanil with another opioid, pethidin. They found no significant difference in systolic, mean and diastolic blood pressure change after laryngoscopy and tracheal intubation where sufentanil (1.5μg.kg-1) was administered intravenously vs. pethidin (1.5mg.kg-1) 3 minutes before laryngoscopy and tachycardia. A difference of more than 20% of basal value was not found between the two groups in this study. Indeed sufentanil didn’t perform better than pethidin in this manner. These authors focused on the time to peak effect to explain the blood pressure control with these two different opioids. As we found hypotension and bradycardia in some cases in our study with selected targets it's better to avoid higher target concentrations for blood pressure control after tracheal intubation specially when opioids would be coadministered with hypnotic agents such as propofol. As we administered targets recommended in the majority of the literature it is unwise to recommend lower target concentrations because of the inability to control blood pressure and heart rate rise. It would be better to reduce the induction speed and limit plasmatic target concentration in the TCI pump setup. These maneuvers could decrease cardiovascular system depression due to a high target concentrations of opioids. With these considerations, sufentanil and remifentanil by means of TCI both could prevent blood pressure rise after tracheal intubation however the more common and sustained bradycardia occurring with remifentanil remains a concern.

**-:**
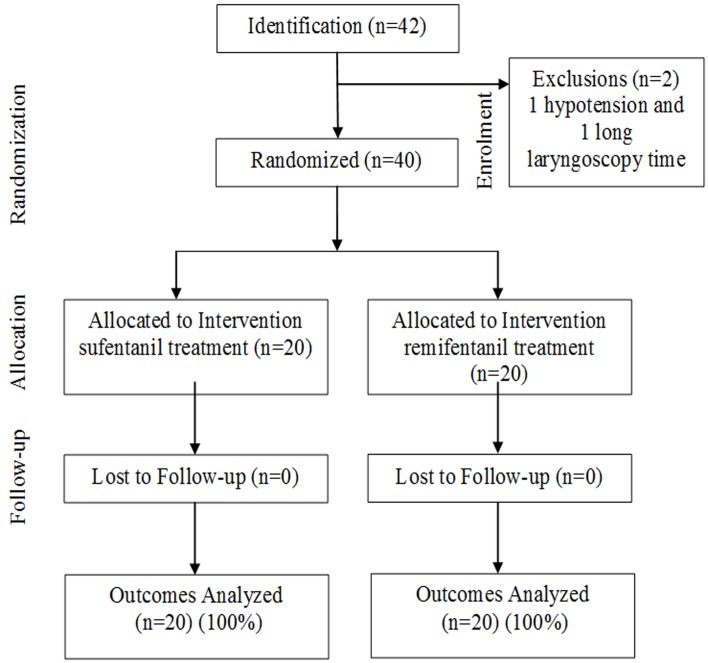
Patients Progress through the trial: CONSORT Flowchart

## Conclusion

Sufentanil produced more common preintubation hypotension than remifentanil in propofol anesthetized patients but this hypotension disappeared sooner than remifentanil after tracheal intubation. On the other hand, remifentanil produced more profound preintubation bradycardia and more sustained bradycardia than sufentanil after tracheal intubation. Target concentration adjustment according to administered hypnotic and increasing the induction time when the TCI technique is used could be recommended to decrease the side effects of opioids during induction. In this manner an increase in blood pressure and heart rate will be prevented when sufentanil or remifentanil are administered with the fewest side effects.
